# Results of Tick-Borne Encephalitis Virus (TBEV) Diagnostics in an Endemic Area in Southern Germany, 2007 to 2022

**DOI:** 10.3390/v15122357

**Published:** 2023-11-30

**Authors:** Philipp Steininger, Armin Ensser, Antje Knöll, Klaus Korn

**Affiliations:** Institute of Clinical and Molecular Virology, Universitätsklinikum Erlangen, Friedrich-Alexander-Universität Erlangen-Nürnberg, 91054 Erlangen, Germany; armin.ensser@fau.de (A.E.); antje.knoell@uk-erlangen.de (A.K.); klaus.korn@uk-erlangen.de (K.K.)

**Keywords:** tick-borne encephalitis (TBE), tick-borne encephalitis virus (TBEV), Germany, diagnostics, antibody detection, intrathecal antibody synthesis, PCR

## Abstract

Tick-borne encephalitis virus (TBEV) is the most important tick-transmitted neurotropic flavivirus in Europe and Asia. Our analysis aimed to investigate the contribution of TBEV-specific antibody detection by serological assays and TBEV RNA detection by real-time PCR to the diagnosis of tick-borne encephalitis (TBE). We analyzed data from 3713 patients from 16 years of laboratory TBEV diagnostics in an endemic area in Southern Germany. During this period, 126 cases of TBE were diagnosed. TBEV-specific IgM ELISA tests showed a high clinical sensitivity (96.8%) and a very high clinical specificity (99.7%). In immunocompetent patients, TBE was reliably diagnosed by detection of TBEV IgM antibodies in serum. Intrathecal TBEV IgG antibody synthesis was detected in 46 of 84 (55%) cases by analysis of paired serum and cerebrospinal fluid (CSF) samples. None of the 87 immunocompetent TBE patients tested had detectable TBEV RNA in serum or CSF. In contrast, in two TBE patients without TBEV-specific antibodies, diagnosis could only be made by the detection of TBEV RNA in CSF. Both patients had previously been treated with the B cell-depleting antibody rituximab. Therefore, in patients with CNS infection and humoral immunodeficiency, it is necessary to include TBEV PCR in the diagnostic approach.

## 1. Introduction

Tick-borne encephalitis virus (TBEV), a neurotropic member of the genus flavivirus, can cause severe infections of the central nervous system (CNS). Morbidity and mortality rates are age-dependent and differ between the viral subtypes [1,2]. Over the last few years, an increasing incidence of tick-borne encephalitis (TBE) has been reported for both endemic and non-endemic European areas [3]. In the region of European Union (EU)/European Economic Area (EEA) countries, Germany reports the third-highest number of autochthonous TBE cases after Czechia and Lithuania [4]. In Germany, the European subtype (TBEV-Eu) of TBEV is maintained in an enzootic cycle between the main vector *Ixodes ricinus* and predominantly small mammals [5]. Almost all human TBEV infections are vector-transmitted, while only a few infections are acquired by consumption of unpasteurized milk or milk products from infected livestock such as goats [6,7]. In Germany, the heterogeneous geographical distribution of TBEV endemic areas and incidence of TBE cases is characterized by a north-to-south gradient, with most human cases occurring in the southern federal states of Bavaria and Baden-Württemberg [8]. Clinical manifestations of TBEV infection range from asymptomatic infection over mild febrile illness to severe meningoencephalitis or encephalomyelitis with substantial potential for long-term sequelae such as post-encephalitic or paralytic syndromes [9,10]. Cases with involvement of the CNS usually have a biphasic course with a first viraemic phase characterized by a non-specific febrile illness followed by a symptom-free interval of a few days before the second phase with acute neurological symptoms and high fever begins [1,2]. Diagnosis based on direct detection of viral RNA using PCR assays is possible only in the first febrile phase but is rarely successful in patients with neurological symptoms. During this second phase, diagnosis relies on the detection of TBEV-specific IgM and IgG antibodies in serum and cerebrospinal fluid (CSF) [2].

Herein, we analyzed the results of 16 years of TBEV diagnostics in a university laboratory in an endemic area in Southern Germany with a focus on the contribution of antibody and PCR testing to the diagnosis of acute TBEV infections in hospitalized patients.

## 2. Materials and Methods

### 2.1. Selection of Patient Samples

Our diagnostic laboratory and the University Hospital Erlangen mainly serve the city of Erlangen and the surrounding counties with a population of approximately 900,000 inhabitants, but as a maximum-care center also the metropolitan region of Nuremberg (3.6 million inhabitants) and beyond. Thus, almost all the patients in this analysis come from the federal state of Bavaria. Overall, the laboratory receives around 70,000 samples per year, among them approximately 700 CSF samples.

All serum samples submitted to our diagnostic laboratory with a request for TBEV IgM and TBEV IgG antibody testing between 1 January 2007 and 31 December 2022 were selected (n = 3992). Furthermore, all CSF samples from this period with a request for TBEV IgG antibody testing and all samples (serum, CSF, and urine) submitted for detection of TBEV RNA were also included in the primary database. Follow-up samples (n = 279) from the same patient were excluded from the statistical analyses but were considered to clarify disease status in patients with suspected acute TBEV infection. Thus, serological results from 3713 patients were included in the statistical analyses.

In addition, residual CSF samples submitted for PCR detection of other neurotropic viruses (mainly herpes simplex virus and varicella zoster virus) were chosen for TBEV real-time PCR. These included all residual CSF samples from routine PCR diagnostics between August 2016 and December 2017 for which a minimum volume of 300 µL was available (n = 892).

### 2.2. Determination of TBEV IgM and IgG Antibodies

For the determination of TBEV IgM and TBEV IgG antibodies, two different assays were used during different periods. Between 2007 and 2015, the Immunozym FSME IgM and IgG ELISA tests (Progen Biotechnik GmbH, Heidelberg, Germany) were run on an ETI-MAX 3000 automated ELISA processor (DIA-SORIN Deutschland GmbH, Dreieich, Germany). Between 2016 and 2022, SERION ELISA classic FSME Virus IgM and IgG assays were run on a Serion Immunomat automated ELISA processor (Institut Virion\Serion GmbH, Würzburg, Germany). Analysis of serum samples for TBEV IgM and TBEV IgG was done according to the manufacturer’s instructions. Equivocal test results were considered negative, and a fourfold rise of TBEV IgG antibodies in follow-up samples was considered indicative of acute infection.

Paired serum and CSF samples submitted for TBEV IgG antibody detection were tested in three different dilutions (1:100, 1:500, and 1:1000 for serum and 1:2, 1:5, and 1:10 for CSF) to measure and compare values within the linear range of the assay over a wide range of IgG levels. The CSF/serum quotient for TBEV IgG was determined by dividing the concentration of TBEV IgG in CSF by the TBEV IgG concentration in serum, taking the chosen dilutions into account. If total IgG and albumin concentrations were available for the respective CSF/serum pair, the TBEV IgG antibody index (AI) was then calculated by division of the TBEV IgG quotient by the total IgG quotient or the Q_lim_ derived from the albumin quotient as described by Siemieniako-Werszko et al. [11]. An AI > 1.5 was considered indicative of TBEV-specific intrathecal IgG synthesis. In cases where no total IgG and albumin values for AI determination were available, but where CSF/serum IgG quotients for herpes simplex virus (HSV) or varicella zoster virus (VZV) were present, TBEV-specific intrathecal IgG synthesis was assumed when the CSF/serum quotient for TBEV IgG was at least twofold higher than the CSF/serum quotients for HSV IgG or VZV IgG.

### 2.3. Detection of TBEV RNA by Real-Time PCR

Sample preparation for the detection of TBEV RNA in routine diagnostics was done using the Qiagen EZ1 Virus Mini Kit on Qiagen EZ1 XL instruments (Qiagen, Hilden, Germany) with an input volume of 200 µL and an elution volume of 90 µL. For the retrospective testing of many CSF samples, sample preparation was performed with the QIAsymphony SP instrument using the QIAsymphony DSP Virus/Pathogen Mini Kit (Qiagen, Hilden, Germany) with an input volume of 200 µL and an elution volume of 60 µL. In both procedures, samples were spiked with a suspension of bacteriophage MS2 equivalent to approximately 2000 copies of the phage genome per sample.

For the detection of TBEV RNA, a one-step RT real-time PCR based on the TBEV-specific primers and probe described by Schwaiger and Cassinotti [12] was developed. Different from the approach described there, we used bacteriophage MS2 as internal control, and therefore, the RT-PCR mix contained primers MS2-TM2F and MS2-TM2R as well as the probe MS2-TM2JOE for the amplification of bacteriophage MS2 RNA as described by Dreier et al. [13]. The AgPath-IDTM One-Step RT-PCR Kit (Thermo Fisher Scientific, Darmstadt, Germany) was used for the RT real-time PCR assays that were run on an Applied Biosystems 7500 Real-Time PCR instrument.

### 2.4. Statistical Analysis

All data were anonymized, and statistical analyses were carried out using GraphPad Prism version 9.5.1 for Windows (GraphPad Software, La Jolla, CA, USA). For the analysis of demographic characteristics and test results, categorical variables were summarized by count and proportion. Differences between groups were analyzed by Fisher’s exact test for dichotomous variables. Differences were considered significant when *p* values were less than 5% (*p* < 0.05).

## 3. Results

### 3.1. Laboratory-Confirmed Acute TBEV Infections

In total, 126 TBE cases were identified between 2007 and 2022 among 3713 patients from whom samples for TBEV diagnostics were submitted. In 124 of 126 (98.4%) patients TBE diagnosis was based on antibody detection. 121 patients had detectable TBEV-specific IgM and IgG antibodies already on admission. One immunocompetent patient presented with an isolated positive IgM test (IgG negative), but the diagnosis was confirmed by seroconversion for TBEV-specific IgG antibodies in a follow-up sample 10 days later. In 2 patients with vaccine breakthrough infections, the first serum sample tested positive for IgG but negative for IgM antibodies against TBEV. However, TBE was confirmed by TBEV IgM seroconversion and a more than fourfold increase of TBEV IgG antibodies in follow-up samples. In contrast, in 2 of 126 (1.6%) patients TBEV-specific IgM and IgG antibodies were completely negative and TBE was diagnosed by the detection of TBEV RNA in CSF.

### 3.2. Temporal Distribution of TBE Cases

**Figure 1:**
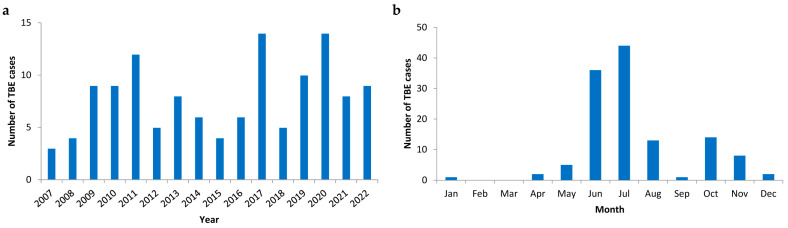
(**a**) Annual distribution of tick-borne encephalitis (TBE) cases (n = 126) and (**b**) monthly distribution of TBE cases (n = 126) between 2007 and 2022.

There was a median number of 8 TBE cases per year with a marked variability ranging from 3 to 14 (Figure 1a). The highest numbers of TBE cases were diagnosed in 2017 and 2020. During the observation period, a moderate increase in the average median number of annual TBE cases was observed from 6 cases to 10 cases. The frequency of TBE cases displayed a pronounced seasonality with a bimodal pattern (Figure 1b) showing a major peak in June and July (representing 63% of all cases) and a minor peak in October and November (representing 17% of all cases). Except for February and March, at least one TBE case was detected in every month.

### 3.3. Demographic Characteristics of TBE Patients

**Figure 2:**
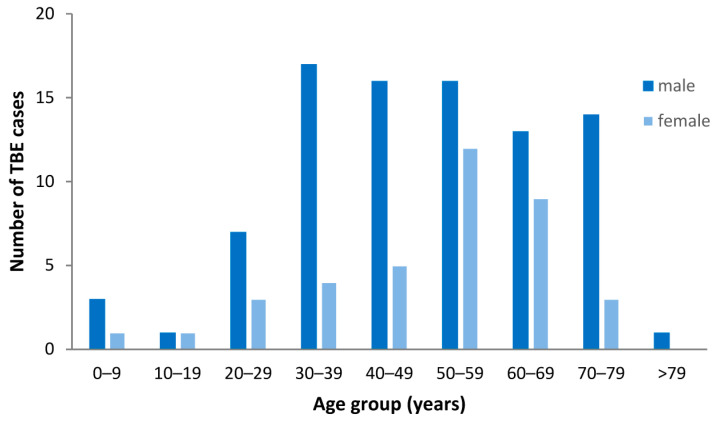
Demographic characteristics of patients with tick-borne encephalitis (TBE) (n = 126) according to sex and age group.

The mean age of the 126 patients was 50 years (range, 3–83 years). 88 patients were male (70%) and 38 female (30%). The number of male patients was higher in all age groups (Figure 2).

The maximum number of TBE cases was observed among 30–79-year-old males (range, 14–17 cases) and 50–69-year-old females (range, 9–12 cases). The largest sex-specific difference of cases occurred in the age groups from 30 to 39 years and from 70 to 79 years with a male-to-female ratio of 4.3 to 1 and 4.7 to 1, respectively. Children and adolescents aged below 20 years accounted for only a minority of cases (6/126; 4.8%) with a smaller predominance of male patients (male-to-female ratio: 2:1). All patients were hospitalized at the departments of neurology (83.3%), internal medicine (11.9%) or pediatrics (4.8%), respectively.

### 3.4. Positive Rates in TBEV Diagnostics

**Figure 3:**
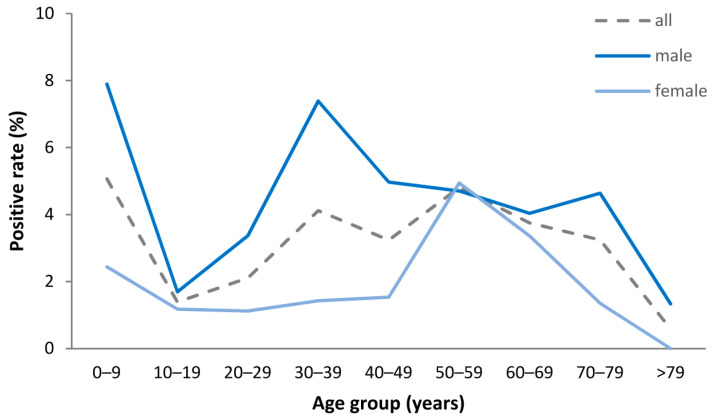
Positive rates in tick-borne encephalitis virus diagnostics according to sex and age group in 3713 tested patients with suspected central nervous system infection.

In 126 of 3713 (3.4%) investigated patients, acute TBEV infection was laboratory-confirmed by antibody detection or real-time PCR. The positive rate of TBE diagnosis varied over the year, positive rates were highest during the first (major) peak in June (8.4%) and July (10%) and lower during the second (minor) peak in October (5.0%) and November (3.0%), respectively. The overall positive rate was higher for male than for female patients (4.6% vs. 2.1%, respectively, *p* < 0.001). The positive rate for males exceeded those for females in all age groups with the highest absolute difference in the age group between 30 and 39 years (7.4% vs. 1.4%, respectively) but nearly equal positive rates between 50 and 69 years (Figure 3). The highest positive rates in adult patients were observed for males between 30 and 39 years (7.4%) and for females between 50 and 59 years (4.9%), respectively.

### 3.5. Clinical Performance of TBEV Antibody Detection in Serum

The performance of the TBEV IgM assay was evaluated for the confirmation or exclusion of TBE in patients with suspected CNS infection. For this purpose, test results for IgM detection in the initial serum sample after hospital admission were analyzed (Table 1).

**Figure 4:**
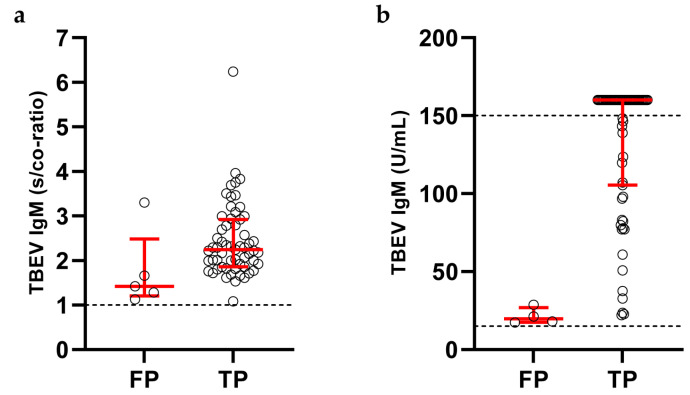
Comparison of tick-borne encephalitis virus (TBEV) IgM test reactivity between false-positive (FP) and true-positive (TP) test results for (**a**) Progen TBEV IgM assay (n = 5 [FP], n = 59 [TP]), (**b**) Virion\Serion TBEV IgM assay (n = 4 [FP], n = 63 [TP]). Each circle represents one sample. Median and interquartile ranges are indicated by red horizontal lines. The lower limit of detection (**a**,**b**) and the upper limit of quantitation (**b**) are indicated by dashed lines. s/co-ratio = sample/cutoff-ratio.

Four of 126 (3.2%) patients with confirmed TBE tested false-negative for IgM in serum, due to prior rituximab treatment (n = 2) or vaccine breakthrough infection (n = 2). False-positive IgM results were observed in 9 of 3587 (0.25%) patients, in whom the clinical course did not match TBE and/or the follow-up sample did not show seroconversion or a significant increase of TBEV-specific IgG antibodies. Among these patients, 4 showed isolated TBEV IgM positivity, while 5 were positive for TBEV IgM and IgG. Except in one case, the reactivity of false-positive TBEV IgM assays was only slightly above the lower limit of detection and significantly lower in comparison to median TBEV IgM levels of true-positive tests (Figure 4). Overall, the sensitivity and specificity of the TBEV IgM assays were 96.8% and 99.7%, respectively. In our cohort, the positive predictive value was 93.1% and the negative predictive value was 99.9%.

### 3.6. Clinical Performance of TBEV IgG Detection in Paired Serum-CSF Samples

**Figure 5:**
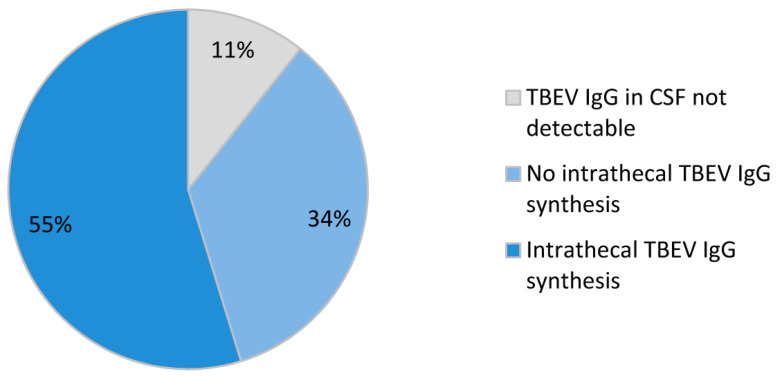
Tick-borne encephalitis virus (TBEV)-specific IgG antibody detection in cerebrospinal fluid (CSF) of tick-borne encephalitis patients (n = 84).

Paired serum and CSF samples for comparative measurement of TBEV-specific IgG antibodies were available in 84 IgG seropositive TBE patients. In 75 of these patients, TBEV-specific IgG antibodies were also detectable in CSF. In 50/75 (67%) cases the TBEV-specific IgG AI was calculated. In the remaining 25/75 (33%) cases only the TBEV-specific IgG CSF/serum quotient could be calculated due to lack of total IgG and albumin values. Intrathecal synthesis of IgG antibodies against TBEV was assumed when the TBEV-specific IgG AI was above 1.5 (n = 31) or, in cases without AI, the CSF/serum quotient for TBEV IgG was significantly (≥2-fold) higher than the CSF/serum quotients for HSV IgG or VZV IgG (n = 15). Intrathecal IgG production against TBEV was observed in a total of 46/84 (55%) patients (Figure 5). In patients with TBEV-specific intrathecal IgG antibody synthesis, the median AI was 2.8 (interquartile range, 2.0–5.8).

### 3.7. Clinical Performance of TBEV Real-Time PCR

In 2 of 126 TBE patients, diagnosis was established by the detection of TBEV RNA in CSF by real-time PCR while TBEV-specific IgM or IgG antibodies were undetectable (Table 2). In both cases, patients had previously been treated with the B cell-depleting antibody rituximab and were therefore not able to produce TBEV-specific antibodies. In one patient, TBEV RNA was also present in urine, while serum samples of both patients tested negative for TBEV RNA. Concentrations of TBEV RNA were very low in all positive specimens displaying cycle threshold values above 33 in the real-time PCR.

Real-time PCR for TBEV RNA was additionally performed in CSF samples of 87 patients with serologically confirmed acute TBEV infection. In none of these samples, TBEV RNA was detectable including one patient with isolated IgM positivity in the very early second phase of TBE.

Furthermore, 892 CSF specimens from patients with suspected viral meningitis or encephalitis were screened for TBEV RNA by real-time PCR. 891 CSF samples were negative for TBEV-RNA, and in one sample the PCR assay was invalid since the internal control was not detectable.

### 3.8. Seroprevalence of TBEV IgG Antibodies in Non-TBE Patients

**Figure 6:**
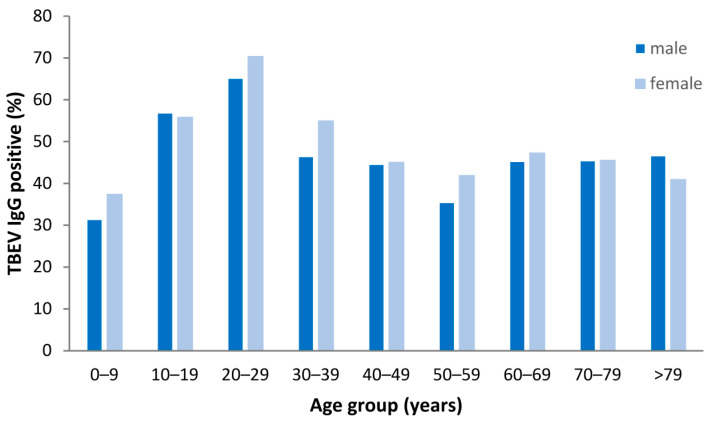
Prevalence of tick-borne encephalitic virus (TBEV)-specific IgG antibodies in serum from 3587 non-TBE patients (1808 males and 1779 females, respectively) according to sex and age group.

In the cohort of non-TBE patients the detected TBEV IgG seroprevalence was 48.2% (1729/3587). The percentage of TBEV IgG seropositive individuals was significantly higher for females in comparison to males (50.5% [898/1779] vs. 46.0% [831/1808], *p* = 0.008, respectively). The highest TBEV IgG seroprevalence rates were detected in the age groups between 20 and 29 years (68.2%; 317/465) and between 10 and 19 years (56.3%; 80/142), respectively (Figure 6). In comparison, TBEV IgG seroprevalence was significantly lower in the age groups above 29 years (45.0%; 1306/2905; *p* < 0.001).

## 4. Discussion

In this retrospective analysis, we analyzed data from TBEV diagnostic procedures in 3713 patients during the period from 2007 to 2022. The focus of the analysis was the description of the demographic characteristics and diagnostic results in the 126 TBE cases that we identified during this period. In our cohort, the submitting hospitals were all located in TBE risk areas, which are defined by the National Public Health Institute in Germany (Robert Koch Institute, RKI) as administrative district areas with a five-year incidence in human cases above 1/100,000 inhabitants [14,15]. For comparison of our local data with German surveillance data, we performed a database query in SurvStat@RKI for all notified TBE cases between 2007 and 2022 (n = 6517) stratified by the distribution of age, sex, and year and week of reporting [16].

Our data showed a marked annual variability of TBE cases with maximum numbers in 2017 and 2020. This was in accordance with German surveillance data showing the highest number of notified TBE cases also in 2020 (760 cases), followed by 2018 and 2022 with 605 and 620 cases, respectively, and 2017 with 503 cases. The peak of the TBE cases in the first COVID-19 pandemic year 2020 might be related to a change in human outdoor behavior due to public health interventions since more frequent outdoor walks have been reported in a case-control study [17]. However, environmental, and ecological factors such as higher tick abundance, might have contributed as well [4]. Interestingly, whereas almost all other notifiable infectious diseases showed markedly reduced case numbers in 2020 compared to the mean of the five pre-pandemic years, TBE was the only notifiable infectious disease in Germany with an increase in the reported case numbers in 2020 [18].

TBE displays a pronounced seasonality with a bimodal distribution due to a variety of environmental and ecological conditions determining tick activity and viral replication as well as human behavior [19]. Our data showed the first major peak of TBE cases in July (35% of all cases) which is in line with the maximum peak in July for all EU/EEA countries, representing 27% of confirmed cases [20]. The second smaller peak was observed in October and November accounting for 17% of all cases in our cohort. However, sporadic TBE cases occur outside the season, even in the winter period. The number of these TBE cases might further rise as a consequence of climate change, due to a shift and increase of seasonal activity of *I. ricinus* as a result of the increasing temperatures [21]. Therefore, in patients with CNS infections of unknown etiology, TBEV diagnostics should always be performed, also outside the characteristic seasons.

The higher rate of TBE cases among males than females in our analysis is in accordance with published data for Europe [20]. The predominance of male patients in our cohort (70%) was even higher compared to the German surveillance data (62% [4030/6517]) [16]. The higher number of male patients might be due to more frequent exposure to tick bites during occupational and recreational behavior in men, while risk perception and use of protective measures might be higher among women [20,22,23]. Since not only the absolute case numbers but also the positive rates in TBEV diagnostics were higher for males than females, we can exclude a sex-related testing bias as a possible reason for the higher incidence rates in men in our cohort.

Analysis of the clinical performance of the TBEV IgM assays in our analysis revealed an excellent specificity of 99.7%. This is in some contrast to recent publications, reporting lower specificities of 80% for the Virion\Serion assay and 90% for the Progen assay [24], and 96% for the Virion\Serion assay [25], respectively. However, these results do probably not reflect the “real-world” performance of these TBEV IgM assays, because the sample size in both studies (69 and 44 true-negative samples, respectively) was too small for a reliable specificity determination. Additionally, in one of these studies [24] more than half of the specificity testing panel consisted of samples from patients with other acute flavivirus infections (denguevirus, zikavirus) or with vaccinations against yellow fever and/or Japanese encephalitis, where cross-reactions may occur. Therefore, our results are most likely more representative of the clinical performance of these tests when used in Germany or other Central or Northern European countries where other flavivirus infections are rare. Regarding sensitivity, there is a discrepancy in the opposite direction, since the two assays were assigned sensitivities of 100% in the two studies mentioned above [24,25], whereas the sensitivity in our samples was only 96.8%. Apart from the fact that the number of true IgM-positive samples in these two studies was also low (18 and 40, respectively), it should be considered that all four false-negative IgM results in our analysis occurred in patients with special conditions (two patients with B cell-depleting therapy and two patients with vaccine breakthrough infections). The rituximab-treated patients were unable to mount a humoral immune response, and in vaccine breakthrough infections, a delayed and weak IgM response in the presence of very high TBEV-IgG levels typical of a secondary immune response is characteristic [26]. Thus, in “normal” acute TBE cases (non-breakthrough infections in immunocompetent patients), the two IgM assays also reached a sensitivity of 100% in our analysis.

Our analysis of 84 paired CSF and serum samples for TBEV IgG in hospitalized patients with acute TBE shows TBEV-specific intrathecal IgG production in more than half of the patients. About 10% of the patients have no detectable TBEV IgG antibodies in the CSF and the remaining patients have detectable TBEV IgG antibodies in the CSF due to diffusion across the blood-brain barrier. In publications presenting data in a similar way, one recent study showed evidence of a TBEV-specific intrathecal antibody production in 5 of 8 patients (62.5%), no TBEV-specific IgG in CSF in 1 of 8 patients (12.5%), and a normal TBEV IgG AI in the remaining 2 patients [24]. An older study from 1997 including 69 patients reported that 41% of patients had detectable intrathecal antibody synthesis on days 0–6 after hospital admission, which increased to 97% on days 7–19 after admission and 98% on days 21 to 62 after admission [27]. This is also comparable to our results, which are based on the first paired CSF and serum samples obtained after admission. Another recent study links a low or delayed intrathecal IgG response to the development of persistent sequelae following TBE but does not report how many patients had a detectable intrathecal IgG response at the different time points in the course of disease [11].

We detected TBEV RNA in the CSF in only 2 of 89 patients with acute TBE which is in accordance with several studies from various European countries (Austria, Switzerland, Slovenia, Hungary, Sweden, Poland) published between 1995 and 2022 [12,28,29,30,31,32,33]. Overall, only 3 of 389 CSF samples tested positive for TBEV RNA in these studies. In our analysis, both TBEV RNA-positive patients had been treated with the B cell-depleting antibody rituximab and were unable to mount a specific immune response against TBEV [34]. Since these two cases appeared within only three months in 2016, we suspected that such cases may occur more frequently due to the increasing use of B cell-depleting antibodies in the therapy of autoimmune diseases. This prompted us to do a large-scale screening of unselected residual CSF samples over a period of 17 months. However, this investigation of 892 CSF samples did not reveal any additional case of previously undetected TBE with positive RNA detection in CSF only. Therefore, these two cases are, together with two other cases from Sweden reported in 2017 [35], the only cases of severe TBE in patients treated with B cell-depleting antibodies reported so far.

While no specific treatment is available for TBE, the disease can be prevented through vaccination. However, despite national vaccine recommendations, vaccine uptake is low in many TBEV-endemic European countries [36]. There is no official data on vaccination coverage for Germany and TBEV-specific IgG ELISA cannot differentiate between vaccine-induced and infection-induced antibodies. However, an ELISA for the detection of IgG antibodies against non-structural protein 1 (NS1) of TBEV has been recently established, which can be used for differentiation between current or past TBEV infection and TBE vaccination [37]. Based on the combination of both serological assays, a recent study evaluated the proportion of TBEV-vaccinated and TBEV-infected individuals in a population from Southern Germany [38]. In total, 57% of all samples tested positive for TBEV-specific IgG while only 5.6% of these samples tested positive for NS1-specific IgG [38]. Therefore, the seroprevalence of TBEV-specific IgG determined in our cohort can be used as an approximation for the vaccination coverage, since the proportion of infection-induced antibodies is only about 10%. Accordingly, the TBEV IgG seroprevalence in our analysis of non-TBE patients (48%) was slightly higher than the self-reported TBE vaccination coverage in Bavaria (40%) [39]. In our analysis, the highest TBEV IgG seroprevalence of up to 68% was found in adolescents and young adults between 10 and 29 years, while the seroprevalence was markedly reduced to 45% in adults above 29 years. Therefore, awareness of the importance of TBE vaccination should be further increased especially in this group.

## 5. Conclusions

Our analysis underlines the high sensitivity and specificity of TBEV antibody detection in serum in a TBEV endemic area and hereby confirms that diagnosis of TBE in immunocompetent patients can rely on the detection of TBEV IgM and IgG antibodies alone. TBEV real-time PCR from CSF has no additional diagnostic value in immunocompetent patients but is necessary for the diagnosis of TBE in patients with humoral immunodeficiency such as a previous treatment with B cell-depleting antibodies like rituximab.

## Figures and Tables

**Table 1:** Tick-borne encephalitis virus (TBEV) IgM assay results in patients with and without tick-borne encephalitis (TBE).

	No TBE	Confirmed TBE	
TBEV IgM negative	3578	4	3582
TBEV IgM positive	9	122	131
	3587	126	3713

**Table 2:** Patients with PCR diagnosed acute tick-borne encephalitis virus infection.

Patient	Age	Sex	Disease	Immunosuppressive Medication	Real-Time PCR Result (Ct Value)		
					CSF	Serum	Urine
**1**	54 y	f	Rheumatoid arthritis	RTX	33.4	negative	negative
**2**	74 y	m	B-NHL	RTX, bendamustine	33.4	negative	35.0

Abbreviations: Cerebrospinal fluid (CSF), cycle threshold value (Ct value), rituximab (RTX), B cell non-Hodgkin lymphoma (B-NHL).

## Data Availability

The data presented in this analysis are available on request from the corresponding author.

## References

[1] Lindquist L., Vapalahti O. (2008). Tick-borne encephalitis. Lancet.

[2] Pustijanac E., Bursic M., Talapko J., Skrlec I., Mestrovic T., Lisnjic D. (2023). Tick-Borne Encephalitis Virus: A Comprehensive Review of Transmission, Pathogenesis, Epidemiology, Clinical Manifestations, Diagnosis, and Prevention. Microorganisms.

[3] Dagostin F., Tagliapietra V., Marini G., Cataldo C., Bellenghi M., Pizzarelli S., Cammarano R.R., Wint W., Alexander N.S., Neteler M. (2023). Ecological and environmental factors affecting the risk of tick-borne encephalitis in Europe, 2017 to 2021. Eurosurveillance.

[4] Van Heuverswyn J., Hallmaier-Wacker L.K., Beauté J., Dias J.G., Haussig J.M., Busch K., Kerlik J., Markowicz M., Mäkelä H., Nygren T.M. (2023). Spatiotemporal spread of tick-borne encephalitis in the EU/EEA, 2012 to 2020. Eurosurveillance.

[5] Michelitsch A., Wernike K., Klaus C., Dobler G., Beer M. (2019). Exploring the Reservoir Hosts of Tick-Borne Encephalitis Virus. Viruses.

[6] Brockmann S.O., Oehme R., Buckenmaier T., Beer M., Jeffery-Smith A., Spannenkrebs M., Haag-Milz S., Wagner-Wiening C., Schlegel C., Fritz J. (2018). A cluster of two human cases of tick-borne encephalitis (TBE) transmitted by unpasteurised goat milk and cheese in Germany, May 2016. Eurosurveillance.

[7] Martello E., Gillingham E.L., Phalkey R., Vardavas C., Nikitara K., Bakonyi T., Gossner C.M., Leonardi-Bee J. (2022). Systematic review on the non-vectorial transmission of Tick-borne encephalitis virus (TBEv). Ticks Tick. Borne Dis..

[8] Topp A.K., Springer A., Dobler G., Bestehorn-Willmann M., Monazahian M., Strube C. (2022). New and Confirmed Foci of Tick-Borne Encephalitis Virus (TBEV) in Northern Germany Determined by TBEV Detection in Ticks. Pathogens.

[9] Chiffi G., Grandgirard D., Leib S.L., Chrdle A., Ruzek D. (2023). Tick-borne encephalitis: A comprehensive review of the epidemiology, virology, and clinical picture. Rev. Med. Virol..

[10] Nygren T.M., Pilic A., Bohmer M.M., Wagner-Wiening C., Wichmann O., Hellenbrand W. (2023). Recovery and sequelae in 523 adults and children with tick-borne encephalitis in Germany. Infection.

[11] Siemieniako-Werszko A., Czupryna P., Moniuszko-Malinowska A., Dunaj-Małyszko J., Pancewicz S., Grygorczuk S., Zajkowska J. (2022). Anti-TBE Intrathecal Synthesis as a Prediction Marker in TBE Patients. Pathogens.

[12] Schwaiger M., Cassinotti P. (2003). Development of a quantitative real-time RT-PCR assay with internal control for the laboratory detection of tick borne encephalitis virus (TBEV) RNA. J. Clin. Virol..

[13] Dreier J., Stormer M., Kleesiek K. (2005). Use of bacteriophage MS2 as an internal control in viral reverse transcription-PCR assays. J. Clin. Microbiol..

[14] Hellenbrand W., Kreusch T., Böhmer M.M., Wagner-Wiening C., Dobler G., Wichmann O., Altmann D. (2019). Epidemiology of Tick-Borne Encephalitis (TBE) in Germany, 2001–2018. Pathogens.

[15] Robert Koch-Institut (2023). FSME: Risikogebiete in Deutschland [TBE: Risk Areas in Germany]. Epid. Bull.

[16] Robert Koch-Institut: SurvStat@RKI 2.0. https://survstat.rki.de.

[17] Nygren T.M., Pilic A., Böhmer M.M., Wagner-Wiening C., Wichmann O., Harder T., Hellenbrand W. (2022). Tick-Borne Encephalitis Risk Increases with Dog Ownership, Frequent Walks, and Gardening: A Case-Control Study in Germany 2018–2020. Microorganisms.

[18] Robert Koch-Institut Infektionsepidemiologisches Jahrbuch Meldepflichtiger Krankheiten für 2020, Page 34/35. Berlin 2021. https://www.rki.de/DE/Content/Infekt/Jahrbuch/Jahrbuch_2020.html?nn=2374622.

[19] Friedsam A.M., Brady O.J., Pilic A., Dobler G., Hellenbrand W., Nygren T.M. (2022). Geo-Spatial Characteristics of 567 Places of Tick-Borne Encephalitis Infection in Southern Germany, 2018–2020. Microorganisms.

[20] European Centre for Disease Prevention and Control (2022). Tick-borne encephalitis. ECDC Annual Epidemiological Report for 2020.

[21] Semenza J.C., Paz S. (2021). Climate change and infectious disease in Europe: Impact, projection and adaptation. Lancet Reg. Health Eur..

[22] Slunge D., Jore S., Krogfelt K.A., Jepsen M.T., Boman A. (2019). Who is afraid of ticks and tick-borne diseases? Results from a cross-sectional survey in Scandinavia. BMC Public Health.

[23] Jepsen M.T., Jokelainen P., Jore S., Boman A., Slunge D., Krogfelt K.A. (2019). Protective practices against tick bites in Denmark, Norway and Sweden: A questionnaire-based study. BMC Public Health.

[24] Reusken C., Boonstra M., Rugebregt S., Scherbeijn S., Chandler F., Avšič-Županc T., Vapalahti O., Koopmans M., GeurtsvanKessel C.H. (2019). An evaluation of serological methods to diagnose tick-borne encephalitis from serum and cerebrospinal fluid. J. Clin. Virol..

[25] Velay A., Solis M., Barth H., Sohn V., Moncollin A., Neeb A., Wendling M.J., Fafi-Kremer S. (2018). Comparison of six commercial tick-borne encephalitis IgM and IgG ELISA kits and the molecular characterization of their antigenic design. Diagn. Microbiol. Infect. Dis..

[26] Dobler G., Kaier K., Hehn P., Bohmer M.M., Kreusch T.M., Borde J.P. (2020). Tick-borne encephalitis virus vaccination breakthrough infections in Germany: A retrospective analysis from 2001 to 2018. Clin. Microbiol. Infect..

[27] Gunther G., Haglund M., Lindquist L., Skoldenberg B., Forsgren M. (1997). Intrathecal IgM, IgA and IgG antibody response in tick-borne encephalitis. Long-term follow-up related to clinical course and outcome. Clin. Diagn. Virol..

[28] Grygorczuk S., Dunaj-Małyszko J., Czupryna P., Sulik A., Toczyłowski K., Siemieniako-Werszko A., Żebrowska A., Pancewicz S., Moniuszko-Malinowska A. (2022). The Detectability of the Viral RNA in Blood and Cerebrospinal Fluid of Patients with Tick-Borne Encephalitis. Int. J. Mol. Sci..

[29] Nagy A., Nagy O., Tarcsai K., Farkas A., Takacs M. (2018). First detection of tick-borne encephalitis virus RNA in clinical specimens of acutely ill patients in Hungary. Ticks Tick. Borne Dis..

[30] Puchhammer-Stockl E., Kunz C., Mandl C.W., Heinz F.X. (1995). Identification of tick-borne encephalitis virus ribonucleic acid in tick suspensions and in clinical specimens by a reverse transcription-nested polymerase chain reaction assay. Clin. Diagn. Virol..

[31] Saksida A., Duh D., Lotric-Furlan S., Strle F., Petrovec M., Avsic-Zupanc T. (2005). The importance of tick-borne encephalitis virus RNA detection for early differential diagnosis of tick-borne encephalitis. J. Clin. Virol..

[32] Saksida A., Jakopin N., Jelovšek M., Knap N., Fajs L., Lusa L., Lotrič-Furlan S., Bogovič P., Arnež M., Strle F. (2018). Virus RNA Load in Patients with Tick-Borne Encephalitis, Slovenia. Emerg. Infect. Dis..

[33] Veje M., Studahl M., Johansson M., Johansson P., Nolskog P., Bergstrom T. (2018). Diagnosing tick-borne encephalitis: A re-evaluation of notified cases. Eur. J. Clin. Microbiol. Infect. Dis..

[34] Steininger P.A., Bobinger T., Dietrich W., Lee D.H., Knott M., Bogdan C., Korn K., Lang R. (2017). Two Cases of Severe Tick-Borne Encephalitis in Rituximab-Treated Patients in Germany: Implications for Diagnosis and Prevention. Open Forum Infect. Dis..

[35] Knight A., Pauksens K., Nordmark G., Kumlien E. (2017). Fatal outcome of tick-borne encephalitis in two patients with rheumatic disease treated with rituximab. Rheumatology.

[36] Kunze M., Banović P., Bogovič P., Briciu V., Čivljak R., Dobler G., Hristea A., Kerlik J., Kuivanen S., Kynčl J. (2022). Recommendations to Improve Tick-Borne Encephalitis Surveillance and Vaccine Uptake in Europe. Microorganisms.

[37] Girl P., Bestehorn-Willmann M., Zange S., Borde J.P., Dobler G., von Buttlar H. (2020). Tick-Borne Encephalitis Virus Nonstructural Protein 1 IgG Enzyme-Linked Immunosorbent Assay for Differentiating Infection versus Vaccination Antibody Responses. J. Clin. Microbiol..

[38] Euringer K., Girl P., Kaier K., Peilstöcker J., Schmidt M., Müller-Steinhardt M., Rauscher B., Bressau E., Kern W.V., Dobler G. (2023). Tick-borne encephalitis virus IgG antibody surveillance: Vaccination- and infection-induced seroprevalences, south-western Germany, 2021. Eurosurveillance.

[39] Erber W., Schmitt H.J. (2018). Self-reported tick-borne encephalitis (TBE) vaccination coverage in Europe: Results from a cross-sectional study. Ticks Tick. Borne Dis..

